# Correspondence

**Published:** 1898-10

**Authors:** John Hudson Grant

**Affiliations:** Acting Assistant Surgeon, U. S. Army.; U. S. General Hospital, Fort McPherson, Ga.


### CORRESPONDENCE.

*To the Editor:*

Sir—When I arrived here, August 14th last, there were 780 patients with an average of sixteen deaths per week from typhoid fever. At this writing we have 650 patients with only six deaths weekly—the advent of cool weather having a very salutary effect in the recovery of the sick men, and in the course of the disease as well. In most of the post mortems held, Peyer's patches were swollen and deeply ulcerated—some with perforation as large as a five-cent piece. In some cases both large and small intestines were involved. The peritoneum, pelvic and mesenteric glands were generally found engorged and the spleen much enlarged and soft. Most of the deaths occurred shortly after their arrival on hospital trains from Tampa and Fernandina.

The treatment most popular here among physicians is stimulation—whiskey, strychnine, and the like, and guaiacol for its antipyretic and antiseptic effect. I have used with good results thymol in capsules to the amount of forty grains a day. We now have seventy-five trained female nurses on duty and to their faithful attendance and nursing many a poor fellow owes his life. Everything wanted and deemed necessary in the treatment and care of these men is liberally provided by the government. The milk bill alone for the month of August amounted to $900 and forty tons of ice was consumed. As soon as men are able to travel and it is deemed safe, they are granted thirty-day furloughs with free transportation to their homes, including a berth in a sleeping-car. After they leave us they are yet in danger, as their appetites are very keen and unless they deny themselves everything, except food of the simplest character, are in great danger of a relapse.

There are a few men here yet undergoing treatment, mostly convalescent, from New York regiments, represented as follows: Second New York, 3; 8th New York, 1; 9th New York, 3; 69th New York, 9; 71st New York, 3. One of the 69th New York is a Buffalo boy—Edgar Leinbaugh, of Walden avenue.

JOHN HUDSON GRANT,
Acting Assistant Surgeon, U. S. Army.

U. S. General Hospital, Fort McPherson, Ga.,
September 13, 1898.

